# Supplementary figures and images for: UV-exposure, endogenous DNA damage, and DNA replication errors shape the spectra of genome changes in human skin
Source: PLoS Genet. 2021 Jan 14;17(1):e1009302. doi: 10.1371/journal.pgen.1009302 (PMC7808690; doi:10.1371/journal.pgen.1009302)

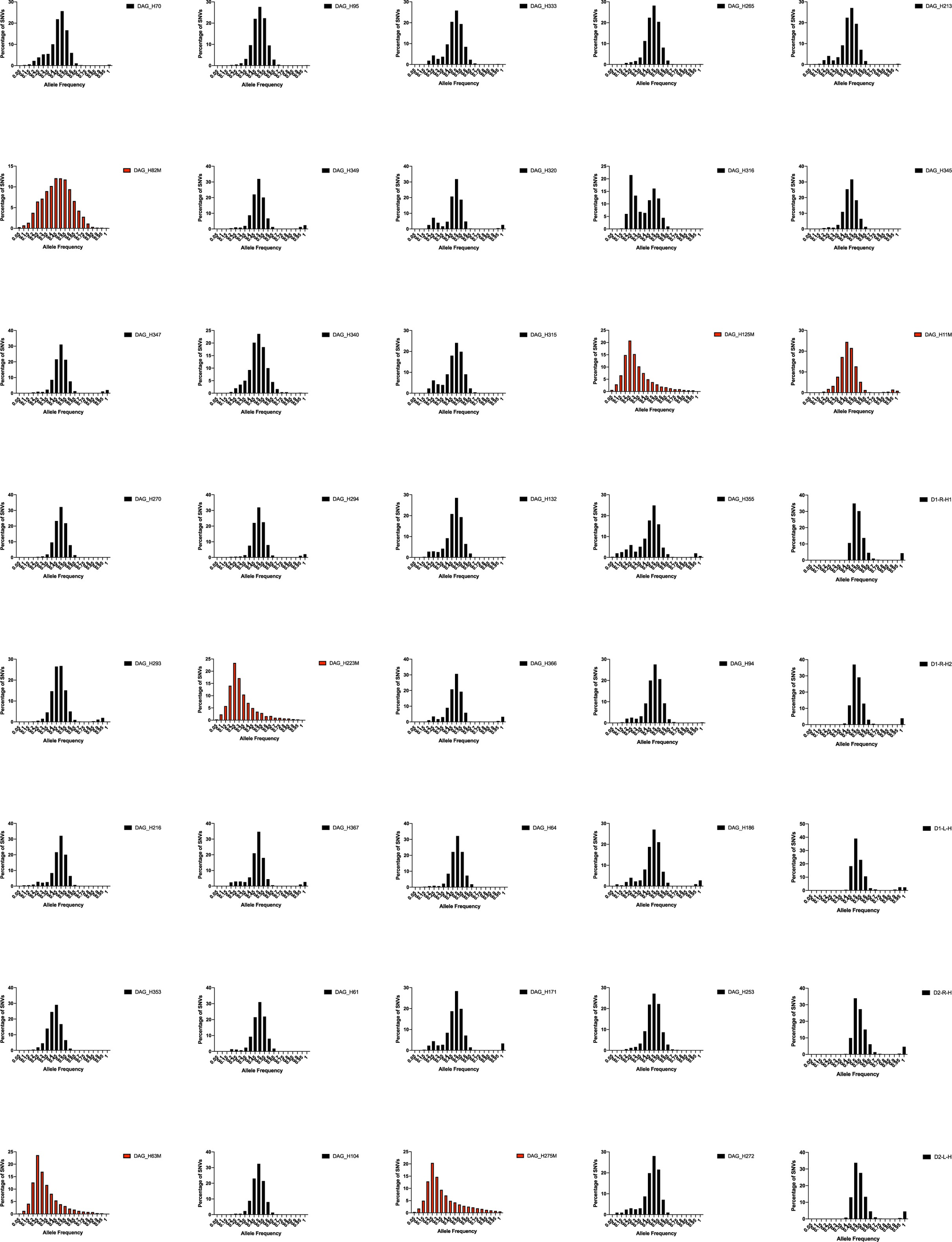

Supplement: S1 Fig — The plots for melanocyte clones are in red. The source data for this figure is in S2 Table. (TIF) [file pgen.1009302.s001.tif]

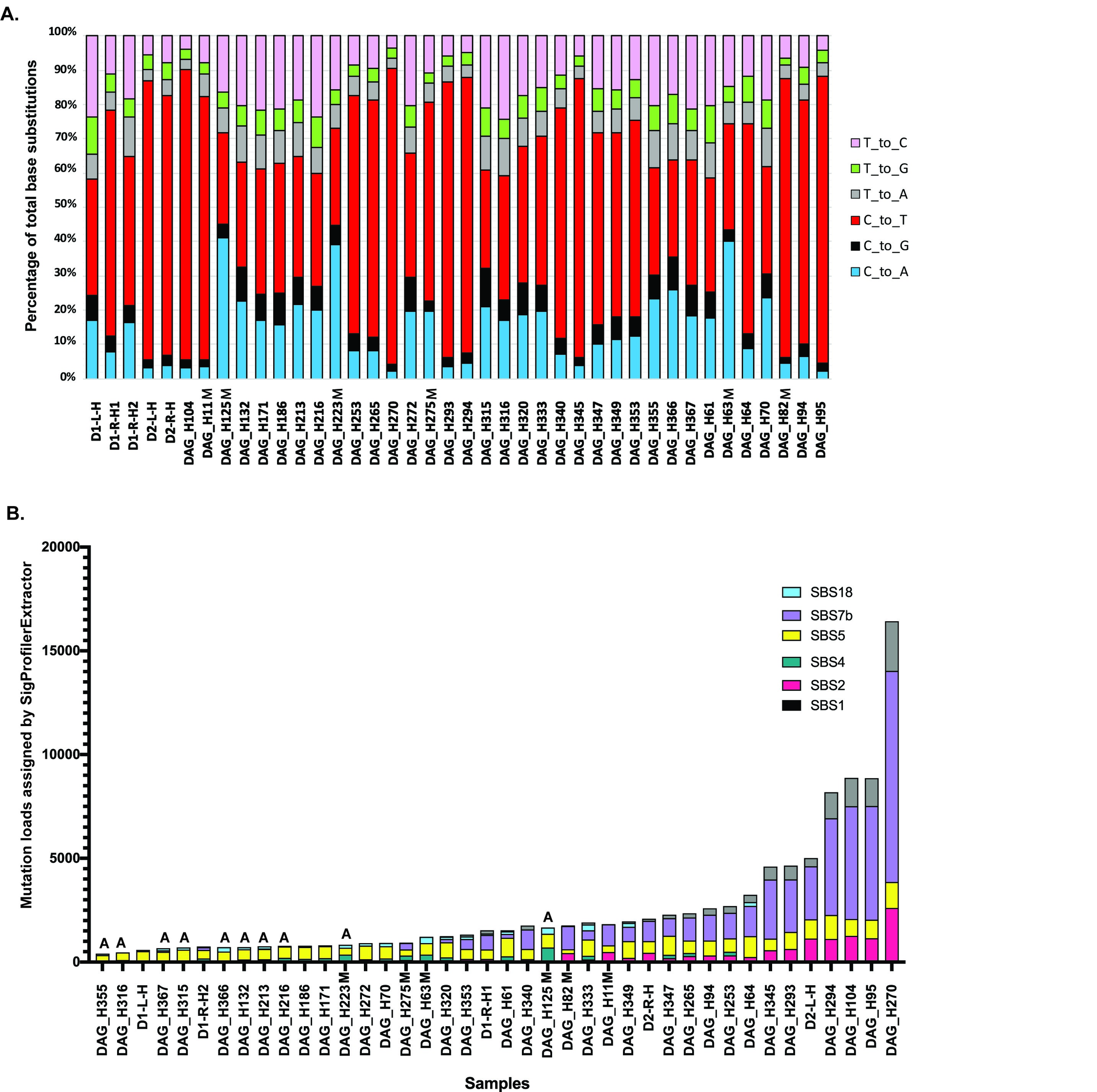

Supplement: S2 Fig — (A)The mutation spectra in each sequenced clone in this study. The melanocyte clones are marked with an “M” in the X-axis. The source data for this figure are in S2 Table. (B) The NMF-derived mutation signature loads as determined by SigProfilerExtractor in each clone sequenced in this study. Samples form African American donors are annotated with an “A” and melanocyte clones are marked with an “M” in the X-axis. The source data for this figure are in S3 Table. (TIF) [file pgen.1009302.s002.tif]

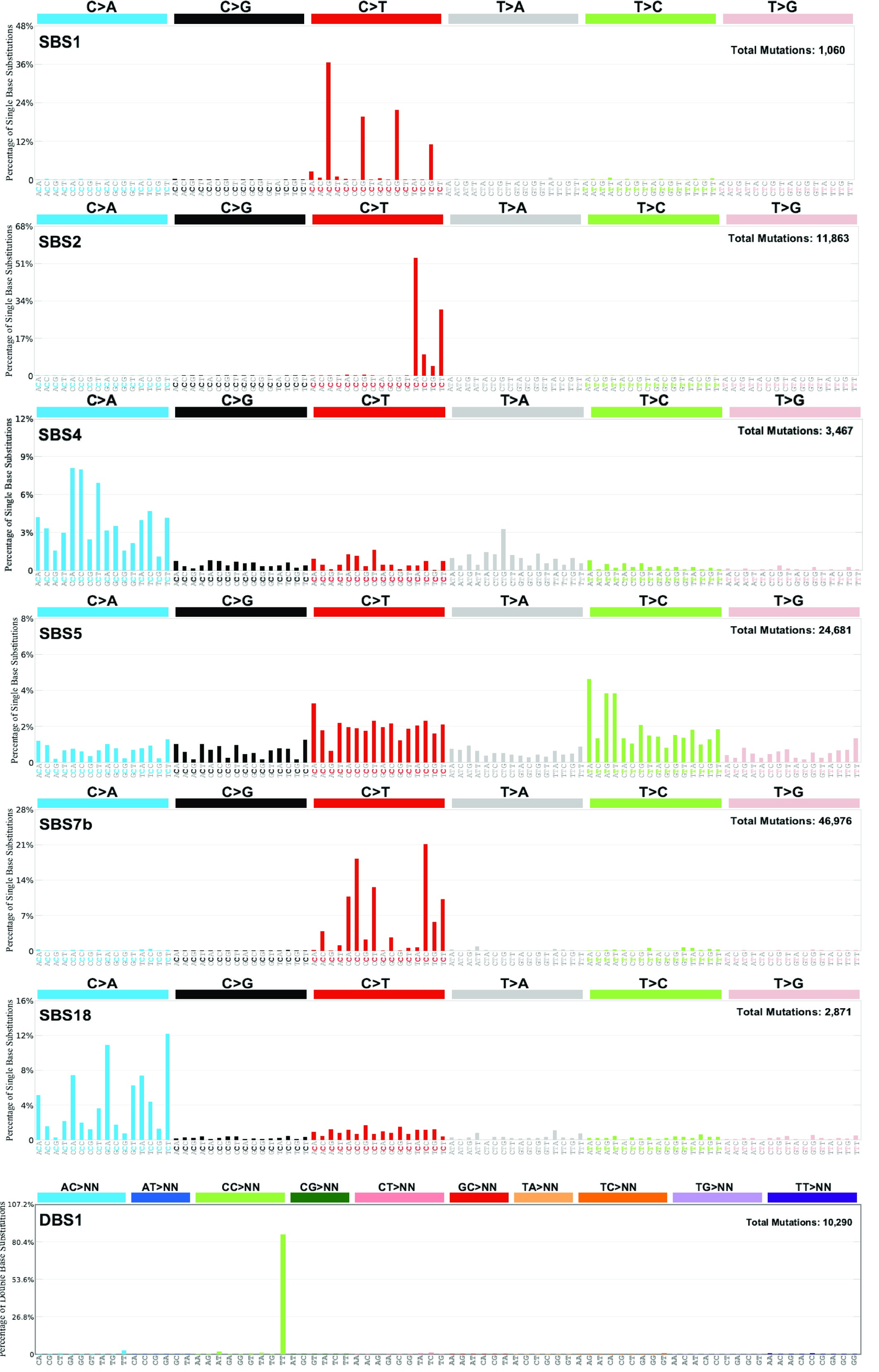

Supplement: S3 Fig — Total mutations corresponding to the signature in the cohort as determined by SigProfilerExtractor are shown. (TIF) [file pgen.1009302.s003.tif]

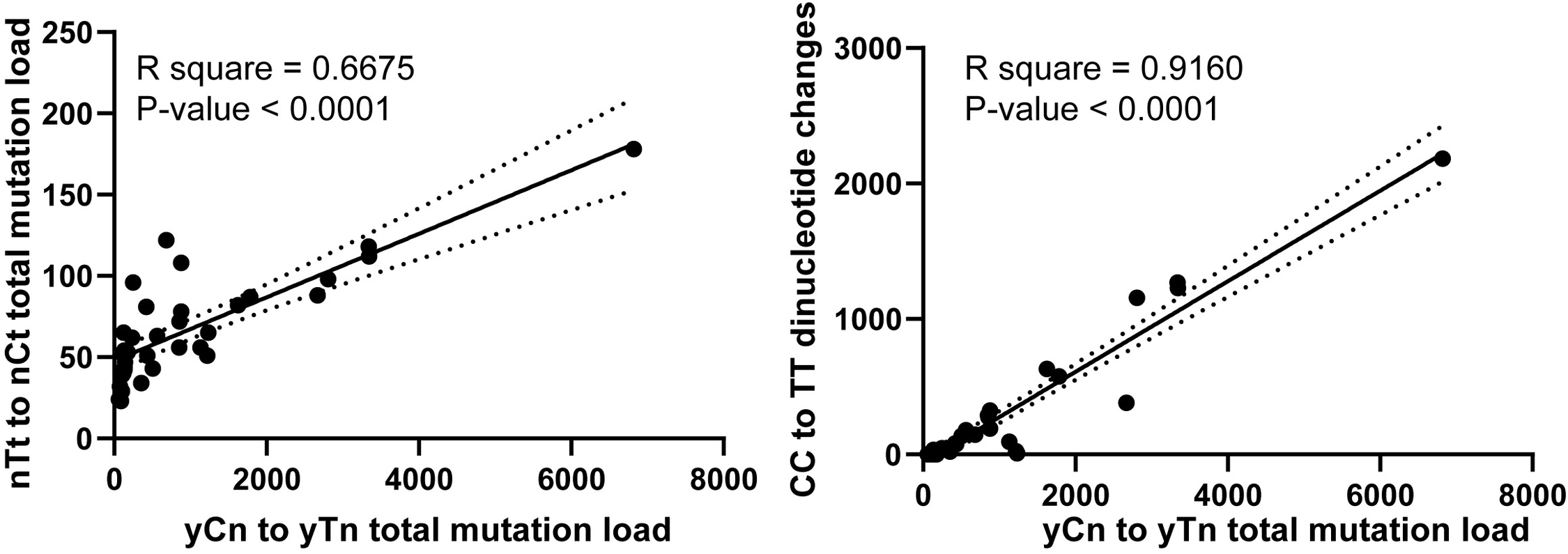

Supplement: S4 Fig — The total nTt➔nCt mutations and the CC➔TT mutations are plotted against the yCn➔yTn total mutation load in the samples. The black inclined line denotes the linear regression of the data, and the dotted black lines denote the 95% confidence intervals. The source data for this figure are in S4 Table. (TIF) [file pgen.1009302.s004.tif]

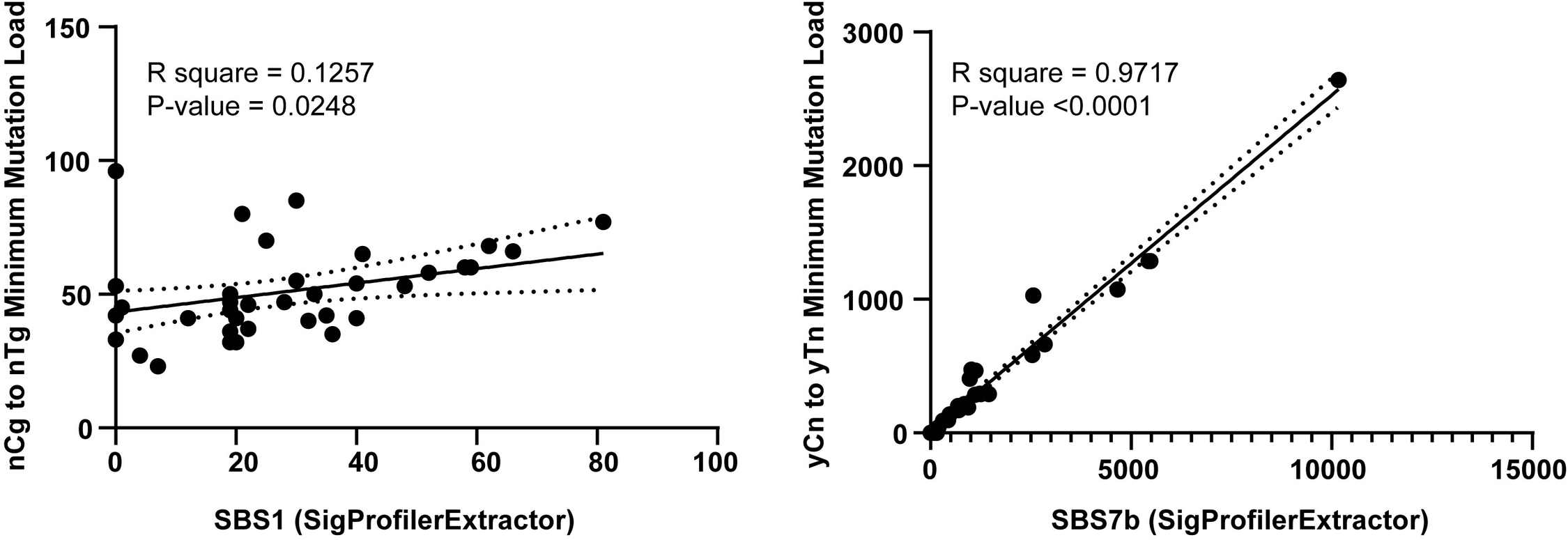

Supplement: S5 Fig — The nCg➔nTg minimum mutation load in each sample is plotted against SBS1-associated mutations as determined by SigProfilerExtractor, and the yCn➔yTn minimum mutation load in each sample is plotted against SBS7b-associated mutations. The linear regression of the data is shown, and the dotted lines denote the 95% confidence intervals. The source data for this figure are in S3 and S4 Tables. (TIF) [file pgen.1009302.s005.tif]

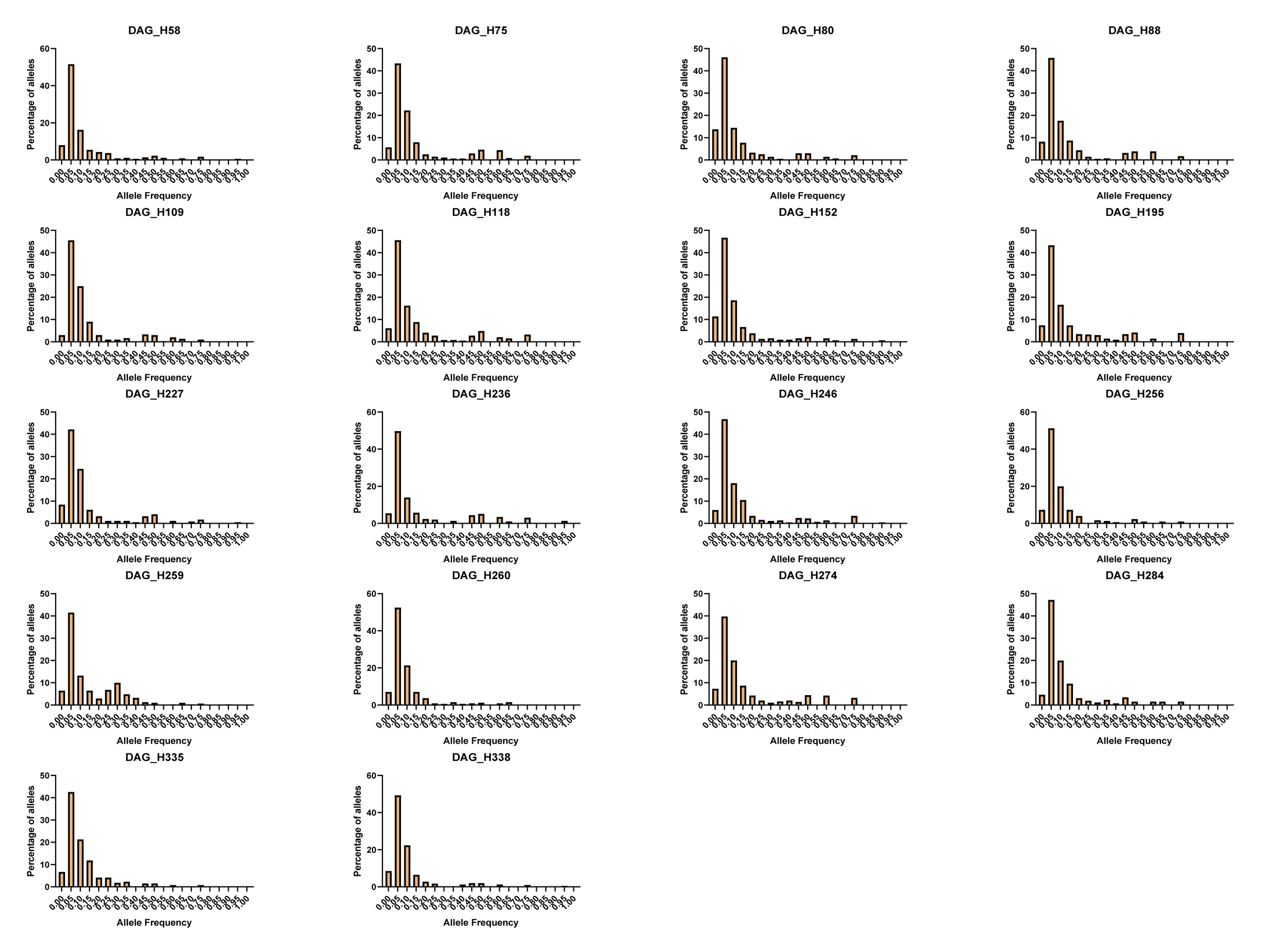

Supplement: S6 Fig — The source data for this figure is in S5 Table. (TIF) [file pgen.1009302.s006.tif]

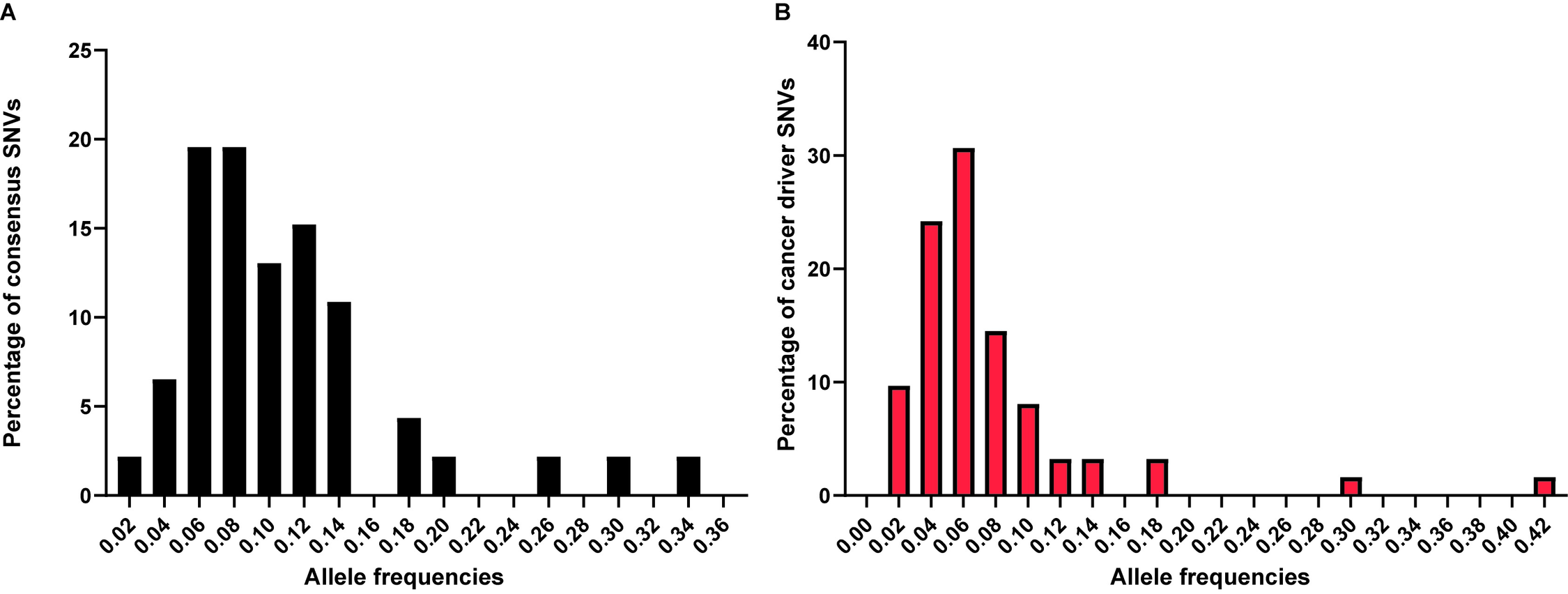

Supplement: S7 Fig — (A) The allele frequencies of the consensus SNVs identified in the bulk and the corresponding clones are shown. (B) The allele frequency distribution of the cancer driver mutations identified in the exome of the bulk samples. The source data for this figure is in S5 Table. (TIF) [file pgen.1009302.s007.tif]

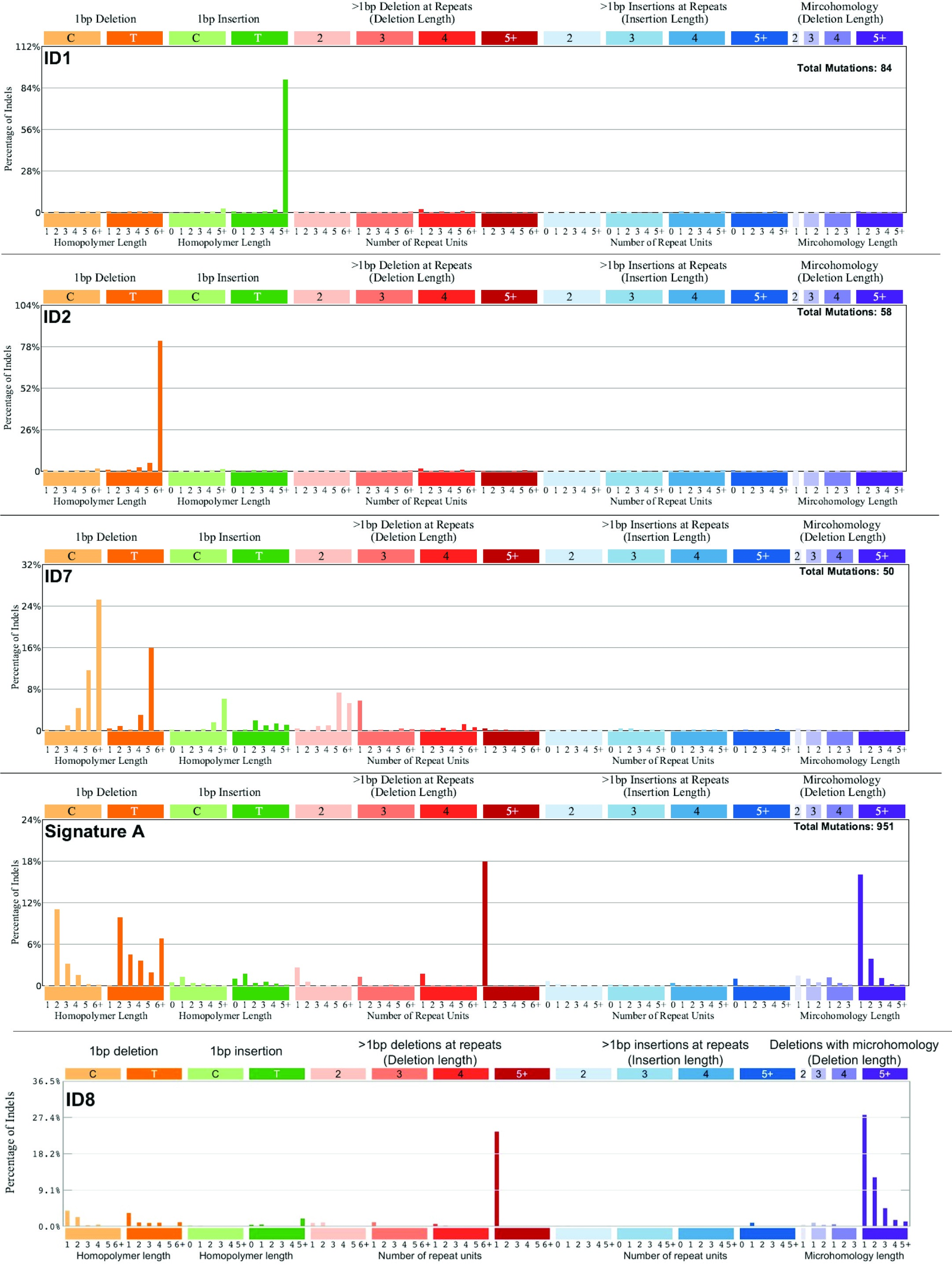

Supplement: S8 Fig — Total mutations corresponding to the signature as determined by SigProfilerExtractor are shown. (TIF) [file pgen.1009302.s008.tif]

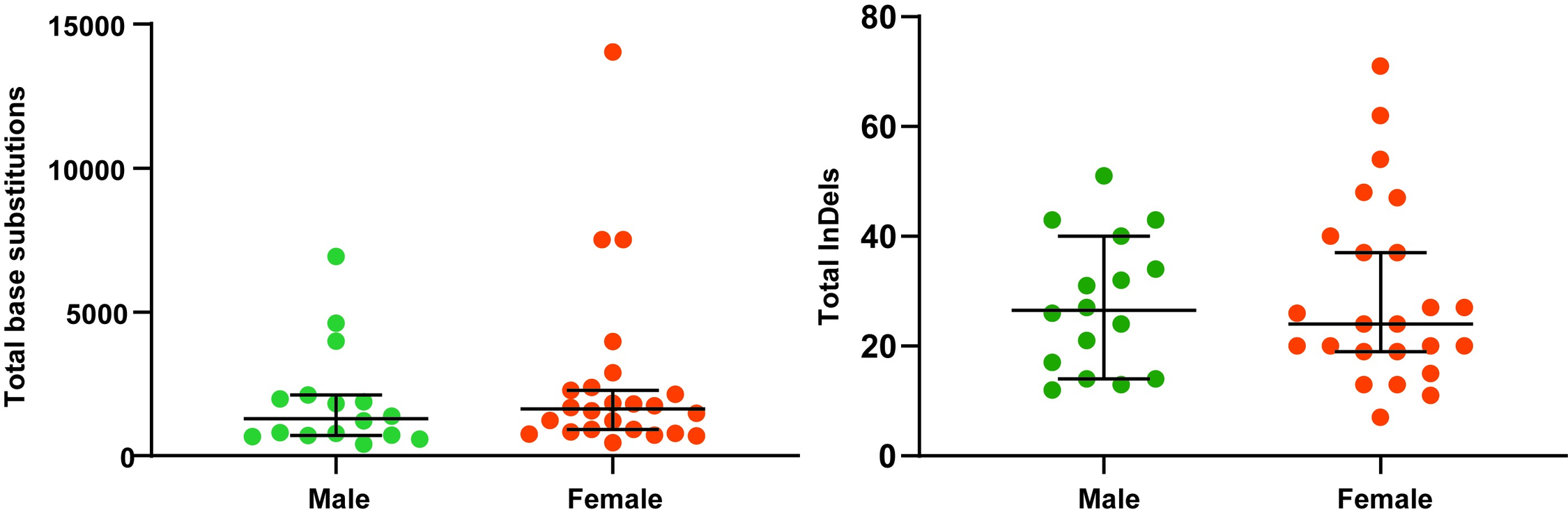

Supplement: S9 Fig — The total base substitutions and the total indels in the clonal lineages derived from males and females in this study are shown. A Mann-Whitney U-test was used to determine if the distribution of mutation and indel load were statistically different between the two cohorts. The P-values for the base substitutions was 0.4041, while the P-value for the indels was 0.9401. The source data for this figure is in S10 Table. (TIF) [file pgen.1009302.s009.tif]
