# Supplementary material for: UV-exposure, endogenous DNA damage, and DNA replication errors shape the spectra of genome changes in human skin
Source: PLoS Genet. 2021 Jan 14;17(1):e1009302. doi: 10.1371/journal.pgen.1009302 (PMC7808690; doi:10.1371/journal.pgen.1009302)
Supplement: Attachment — Submitted filename: 2020_Saini_Response_to_Reviewers_V3.docx [file pgen.1009302.s021.docx]

PGENETICS-D-20-01306 – Point by point response

We thank reviewers for careful analyses of our manuscript and for useful suggestions and comments, for which we have accounted in the revision and described below. Modifications to the original submission are shown by color in this response and are marked by track changes in the marked-up copy file of the manuscript. In our responses, all references to the locations in the manuscript are shown as line numbers in the marked-up revised version unless noted otherwise. (Note that line numbers in the clean revised version are different, because line-numbering software counts ever tracked change as a separate line)

**Reviewer 1**

In the manuscript the authors describe their study of somatic mutations in the normal skin of multiple individuals. For this they rely on cell cloning, i.e., single cells are cultured until colonies are of sufficient size to be sequenced without (or with little) DNA amplification. There were a few previous and similar studies conducted and cited in this manuscript. The novel aspects of this study are: 1) cloning and studying mutations in melanocytes; 2) using samples from different races; 3) using samples from different sexes; 3) a wide age range of participants donated skin samples; 4) analysis that spans beyond just SNVs and includes indels and SVs.

The study reports several significant, new, and interesting findings, of which for me the most interesting were:

* support of UV-induced mutations from by identified indels;

* the experimental evidence that dark skin has protective effect against UV-induced mutations.

I only have a few suggestions on how to improve the manuscript, as well as some minor questions/concerns.

*Response:* We thank the reviewer for their thoughtful comments and suggestions. We have addressed these below.

1. The threshold of allele frequency for somatic variants in clones “within 45% and 55%” seems quite stringent. Could the authors estimate their sensitivity for discovering the variants from founder cells in a clone?

*Response:* We appreciate the Reviewer’s concern regarding the stringency of our allele frequency cutoffs. In order to clarify the reasoning behind this allele frequency filtering criteria in identifying variants in the founder cells in a clone we have now added the following text and Suplpementary Tables to the manuscript –

a. Lines 190 – 199 – “We also analyzed the allele frequencies of all somatic base substitutions in the clonal lineages. All fibroblast clonal lineages demonstrated a peak of mutation calls at the 45% to 55% allele frequencies, indicating that these samples were clonal (FigS1 and S2a Table). We did not see such a peak in the whole-genome amplified melanocyte clones which could reflect uneven genome amplification and localized genome duplications during the whole-genome amplification step. Nonetheless, only analyzing heterozygous mutation calls within the 45% and 55% allele frequencies and homozygous mutation calls at >90% allele frequencies allows us to estimate the minimum accurate number of somatic mutations in the founder cells. Mutations that accrue during culture and/or polymerase errors during whole genome amplification are expected to not be clonal and have allele frequencies <45%.”
b. S1 Figure – Allele frequency distribution in all clones sequenced demonstrating the peaks at 45% to 55% and >90% allele frequencies.
c. S2a Table – The allele frequency distribution of all the SNVs identified in the clones.

2. Can the authors estimate an average increase in non-UV-induced mutations with age?

*Response:* In order to estimate the average increase in both UV and non-UV-induced mutations with age, we have now added the calculations for slopes in Figures 1, 2 and 3.

3. Figure 3B. It is common to show the distribution of indels using negative values for the length of deletions. Please consider this.

*Response:* We have now modified the figure to show deletions using negative values.

4. It was unclear which variants were analyzed for cancer drivers. Were there any mutations discovered in clones and in bulks that were predicted to be a driver?

*Response:* In order to further clarify which variants were analyzed for cancer drivers, we have now modified the text as follows:

Lines 286-289 – “We also annotated all the SNVs in all whole-genome sequenced clones and whole-exome sequenced bulk tissues for functional effects. All non-synonymous SNVs, stop gains, start or stop loss SNVs in the clones and bulk tissues were further analyzed using the Cancer Genome Interpreter [39] to determine if these were potentially cancer drivers.”

5. Can the authors comment about breakpoint features of SVs in CFS and out of CFS? Were there any differences?

*Response:* To identify and comment on any differences in the SVs and the breakpoints between those that were in CFSs and those that were out of CFSs, we have added the following to the manuscript:

a. S11 Table has now been annotated with the presence of microhomologies at the breakpoints.
b. We also performed a Fisher’s exact test to identify if there were any differences between the usage of microhomologies at the breakpoints that overlap CFSs and those that do not.
c. Figure 5c has been added which depicts the types of rearrangements that either overlap or do no overlap CFSs.
d. We have now also added the following text to the manuscript –

Lines 483-490 – “Interestingly, we did not see any difference in the types of structural variants that overlap and those that did not overlap CFSs (Fig 5C). Moreover, we determined the use of microhomology at the breakpoints to identify a role of microhomology mediated repair of DSBs at CFSs. Of the 120 rearrangements, only 15 rearrangements contained microhomology at the breakpoints (6 overlapping CFSs, and 9 not overlapping CFSs). These regions of microhomology were small and ranged from 2 to 3 bases. A Fisher’s exact test demonstrated no significant bias in the use of microhomology between the variants that overlapped CFSs versus those that do not (P-value 0.41) (S11 Table).”

6. Could the authors comment on the possibility of SVs in the CFS being the results of culturing fibroblasts before deriving clones? I wonder whether CFSs mentioned in the text may have been determined as such from cultured fibroblasts?

*Response:* We are cognizant of the concern that we cannot rule out that the SVs in the analyzed samples were a result of culturing fibroblasts prior to deriving clones. Due to difficulties in analyzing SVs in bulk cultures, we are unable to verify whether or not the detected SVs were present in the bulk population. However, the low number of passages prior to deriving clones as well as the similarities in the SVs in our study to other similar studies is indicative of the SVs likely being present in the skin cells prior to culture.

As such, we have now added the following statement to the manuscript –

Lines 656-662 – “Due to the low number of variants, we cannot rule out that some of the structural variants may have been generated due to a rearrangement in during the first few cell divisions of the founder cell in culture. However, we think this is unlikely as these cells were passaged less than 3 times before the generation of a clonal lineage. Moreover, the number and types of variants are similar to those detected in previous studies [69-71] indicating that the structural variants detected in our work were likely present in founder cells.”

7. Line 346: Should there be a letter after the first stated CFS “FRA7”.

*Response:*  The statement (Line 460 in the revised manuscript) has been edited and “FRA7” has been removed from the line.

**Reviewer 2**

In this work Saini et al extend their previous study (Saini et al, PloS Genetics 2016) on the whole genome sequencing of clonally expanded skin fibroblasts sampled from anatomical sites presumed to have experienced a range of UV exposure. The previous work considered six sampling sites from one donor and four sampling sites from a second donor, both donors of primarily European ancestry. This new work re-analyses the hip-derived samples from the existing data but substantially adds to it expanding the number of donors to 21, including ethnic diversity as a sampling metric and including a limited comparison of mutation patterns between skin fibroblasts and melanocytes.

Analysis includes the quantification of single base substitution signatures, double base changes, indels and structural changes. Several of the results are confirmatory, but importantly so, for example the UV signature mutation load not correlating with patient age and the consistent detection of substantial UV signatures even in sites that are mostly shielded from the sun. However, comparisons of mutation spectra and loads for non-cancerous samples, between ethnicities is to my knowledge novel at the whole-genome multiple sample scale. This reveals a striking reduction in UV signature mutation load for African American samples compared to European – not an unexpected result but important validation of expectation. The authors also find that >4bp deletions also show a relative enrichment in European ancestry skin, suggesting a UV contribution to these multi-basepair deletions. The other key finding is the high frequency of chromosomal rearrangements with breakpoints localised approximately to common fragile sites – again building on earlier observations of the same group but much more solid in this expanded study.

Overall the manuscript is well thought out and presented. The results do provide new insights and the primary data generated is likely to be of interest to multiple groups working on the mechanisms of mutagenesis and those studying skin cancer. To the extent practically possible with human sample data, the experiments are generally well controlled and conclusions justified by the evidence presented. Probably the most important aspect of this work is the comparison of mutation spectra and loads for skin from patients with dramatically different levels of naturally occurring melanin, which is expected to protect from UV damage.

A fundamental limitation to the interpretation of these results is that UV exposure per donor is unmeasured and not controlled for. Being hip derived samples the assumption is that these are sites predominantly shielded from the sun, but as such, small differences in exposure (e.g. one bad sunburn) could strongly distort the total lifetime exposure to UV. I think that a caveat/qualification to this effect should be more clearly stated in the discussion.

*Response:* We thank the Reviewer for complementing our study design and highlighting the novel aspects of our work. We agree with the Reviewer that subtle differences in UV-exposure can have strong impacts on the total UV-mutation loads accumulated over the lifetime of the donors. We have now added this statement to the discussion.

Lines 539-543 – “Since we are measuring mutation load in sun-shielded skin cells, as such, even intermittent UV-exposure due to clothing and lifestyle choices of the participants during their lifetimes are likely to lead to the formation of UV-induced DNA damage and impact the lifetime accumulation of UV-induced mutation load in hip-derived cells.”

Other comments:

While I am supportive of publication, I suggest the following points could be addressed where practical.

1. Currently each donor is classified as “White” or “Black or African American”. Within both of these groups there is likely to be a range of skin pigmentation in non-sun-exposed sites (hips). Were measures of that baseline pigmentation taken? It would be useful to report those measures and correlate mutation load/spectra rather than the binary classification of ancestry.

*Response:* Although measures of baseline skin pigmentation would indeed be useful to correlate with mutation loads and spectra, unfortunately the Environmental Polymorphisms Registry at NIEHS does not currently obtain such measurements during recruitment or recall of the donors. As such, we cannot comment on the range of skin pigmentation in our donors and can not correlate this feature with UV-induced mutation loads.

2. Figure 4 plots include melanocyte clones (the “White” outlier with ~14k SBS mutations must be DAG_H275) but these aren’t discriminated in these plots as they are in figures 1 to 3. They should be indicated, particularly when they represent outlier data-points. Do the significant differences noted in these plots remain significant after excluding the melanocyte clones?

*Response:* We have now marked melanocyte clones in Figure 4. In addition, we have analyzed the data with melanocytes excluded. The differences between the mutation loads in the White donors and the African American donors remained significant even upon removal of the melanocytes from the analysis. This analysis is presented in S10b Table and in the text as –

Lines 416-420 – “In order to avoid skewing of the data due to differences in sequencing methodologies used for melanocytes and fibroblasts in our samples, we also calculated P-values for each of the cohorts using a two tailed Mann-Whitney test after excluding the data from the melanocytes. Even in this data set, we were clearly able to detect an increase in UV-induced mutations in White donors as compared to African American or Black donors (S10b Table).”

3. A useful complement to the existing plots would be a plot summarising: (a) SBS mutation load per clone,

(b) contribution of mutation signature contribution per clone like Fig S1 but with a richer set of signatures such as COSMIC,

(c) annotation of ethnicity and cell type (fibroblast/melanocyte) per clone. Such a plot, possibly rank-ordered by total mutation load, would be an informative summary of the key data generated and would make apparent some results that appear to be masked in the current presentation. For example the clone DAG_H275 has a high SBS load and from Fig S1 looks to be dominated by C→T substitutions consistent with UV exposure, but then comparison to Fig 4a (highest purple value to highest red value) and mutation counts in table S2 indicate that this clone must dominated by rCn to rTn mutations (COSMIC signature SBS2). Is this an outlier amongst melanocyte clones?

*Response:* We have added a graph to S2 Fig to account for the changes suggested by the Reviewer above.

We would also like to thank the reviewer for pointing out the outlier data in our analysis. Unfortunately, upon review we realized that Fig S1 had an error in annotations of the clone names. The error has now been corrected. As such, clone DAG_H275 no longer has an exceptionally high SBS load.

The outlier in our samples that the Reviewer is mentioning is DAG_H270. The difference between the donor’s total mutation loads vs UV-specific mutation loads is due to the setup of analysis we used. We report the minimum estimate of mutation load (“Minimum Mutation Loads”) for mutations conforming to the most characteristic trinucleotide motif and caused by a motif-preferring mutagenic mechanism (e.g. yCn🡪yTn changes by UV light), as opposed to “Total Mutation Loads” pertaining to a complete set of motifs within given mutation signature. This allows to highlight individual samples with statistically significant enrichment with signature-defining oligonucleotide motif even if this motif has the minor presence within a signature (e.g., nTt🡪nCt motif within UV signature). Strong correlation between “Minimum Mutation Loads” for this minor motif and major UV motif yCn🡪yTn (Figure S4) in relatively small number of samples underscores the value of this approach. Since the calculation of each sample-specific “Minimum Mutation Load” is prorated by enrichment with trinucleotide-specific mutations over expected number caused by random mutagenesis, this number is less than the total number of this trinucleotide motif mutations in a given sample.

4. Figure 3b. It is difficult to see the individual histogram bars, especially at the left edge. Suggest that this plot would be more informative if points were used rather than histograms and the x-axis shown on a log scale (in addition to the y-axis being kept on a log scale).

*Response:* We have edited the figure as suggested by the Reviewer as well as incorporated changes suggested by Reviewer 1 above.

5. Figure S2. X-axis annotations are not clear (partially overlapped by subsequent panels).

*Response:* We have fixed the figure to avoid overlaps between the panels.

6. Code availability: “The R-code for analysis of the trinucleotide-specific mutation signatures is the same as used in [8] and will be provided on request.” For efficient and long-term reproducibility code, especially that being used across multiple publications, it should be in a pubic repository such as GitHub, Figshare, Zendo or an institutional repository that provides a DOI.

*Response:* We agree with the Reviewer and have made our code for analysis of trinucleotide signatures available publicly via GitHub. Now the “Code availability” section reads as:

Lines 725-726 – “The R-code for analysis of the trinucleotide-specific mutation signatures is the same as used in [8] and can be accessed via <https://github.com/NIEHS/P-MACD>”

7. Data availability: In the additional information front section of the submission, the authors state “Yes - all data are fully available without restriction”. This should also be clearly stated in the manuscript. I would also suggest that the work will be more widely used and cited if the full list of somatic mutation calls (e.g. MAF files filtered for germline variants) can be made available without the requirement for DAC approval, which although an important process for limiting access to potentially personally identifiable germline genotypes, is unnecessary limiting for somatic mutations.

*Response:* As per the rules for dbGAP submission of human variant calls, we are unable to freely provide the MAF files without the requirement for DAC approval. However, to enable the readers to easily locate the data, we have now added the following statement in our methods –

Lines 646-648 – “The mutation calls for both whole exome and whole-genome sequencing have been organized as MAF files in the TCGA format and have been submitted to dbGAP study phs001182.v2.p1.”

We note that due to dbGaP process the version of the study containing MAFs will go live synchronously with publication.

**Reviewer 3**

The manuscript by Saini et al. provides a very interesting analysis of mutation signatures in primary skin cells in donors of different ages and races. As such, it is of great interest to the scientific community. I have several major and minor points for authors to address.

*Response:* We thank the Reviewer for reading and critiquing our manuscript as well as pointing out that it is of interest to the scientific community. We have addressed the major and minor points below.

Major points

1. How did sequencing errors affect the results? How did whole-genome amplification, and potential errors and biases, affect the results? Please justify the choice of 45%-55% and 90% threshold for allele frequencies used. Were simulations used to derive these?

*Response:* We appreciate the Reviewer’s concern regarding sequencing errors and errors during whole genome amplification impacting our data. To further clarify the importance and the choice of the allele frequency filters in enabling us to avoid such errors in our data analysis, we have now added the following to the manuscript –

a. Lines 190-199 – “We also analyzed the allele frequencies of all somatic base substitutions in the clonal lineages. All fibroblast clonal lineages demonstrated a peak of mutation calls at the 45% to 55% allele frequencies, indicating that these samples were clonal (FigS1 and S2a Table). We did not see such a peak in the whole-genome amplified melanocyte clones which could reflect uneven genome amplification and localized genome duplications during the whole-genome amplification step. Nonetheless, only analyzing heterozygous mutation calls within the 45% and 55% allele frequencies and homozygous mutation calls at >90% allele frequencies allows us to estimate the minimum accurate number of somatic mutations in the founder cells. Mutations that accrue during culture and/or polymerase errors during whole genome amplification are expected to not be clonal and have allele frequencies <45%.”
b. We have also added S1 Figure and S2a Table depicting the allele frequency distribution in each clonal lineage sequenced.

2. The finding that UV-induced mutation load is independent of age is counter-intuitive. Please provide an explanation behind this unexpected result. Also, how do the authors explain that UV-based substitution signatures were prominent in many samples obtained from sun-shielded skin?

*Response:* We agree with the Reviewer and have amended the text as follows to explain this result.

Lines 537-545 – “Interestingly, although non-UV mutations (nCg🡪nTg and indels at homopolymeric repeats) increased with the ages of the participants, we did not see a similar correlation between UV-exposure-induced mutations and ages of the donors. Since we are measuring mutation load in sun-shielded skin cells, as such, even intermittent UV-exposure due to clothing and lifestyle choices of the participants during their lifetimes are likely to lead to the formation of UV-induced DNA damage and impact the lifetime accumulation of UV-induced mutation load in hip-derived cells. Thus, the absence of a correlation between age of the donors and the UV-induced mutation load might be due to differences in overall accumulation of UV-exposure across the lifetime of different donors.”

3. The paper should be read by a native speaker of English to fix some minor grammar issues.

*Response:* We have had a native speaker of English read our manuscript and fixed minor grammar issues along with those suggested by the reviewer below.

4. The authors should provide all their code on GitHub.

*Response:* We have now made our code available on GitHub and modified “Code availability” section accordingly.

5. The authors should provide a file with all the genetic variants they identified including their genomic locations for all the samples.

*Response:* Unfortunately, the rules of dbGAP prevent us from providing a file with all the genetic variants we identified in our study publicly as these are identified as controlled-access data. These files can be downloaded from dbGAP upon request and authorization by the data access committee. To enable the readers and members of the scientific community to easily identify the dbGAP study linked to our data, we have now added the following statement in our manuscript –

Lines 646-648 – “The mutation calls for both whole exome and whole-genome sequencing have been organized as MAF files in the TCGA format and have been submitted to dbGAP study phs001182.v2.p1.”

We note that due to dbGaP process the version of the study containing MAFs will go live synchronously with publication.

6. Can the authors speculate about the applications of their study with respect to the increased risk of skin cancer in individuals with high UV exposure?

*Response:* We have now added a section to the discussion in accordance with this suggestion.

Lines 558-566 – “We also demonstrated here that UV-associated mutation load is decreased in skin cells from African American donors as compared to White donors. We hypothesize that this effect may be due to the protective effects of melanin on UV-induced DNA damage. In agreement with the decreased mutation load, are the lower rates of skin cancer in African Americans. While skin cancer accounts for up to 35 - 45% of all cancers in Caucasians [52], it only accounts for 1 – 2% of the neoplasms in African Americans [53-55]. Moreover, the impact of UV-exposure as a risk factor for skin cancers is decreased in African Americans as compared to Caucasians [55, 56]. These observations imply that lowered mutation burden due to UV-radiation is indicative of the lower risk of UV-induced skin cancer in African Americans.”

Minor Points

1. The title should read 'UV exposure, endogenous DNA damage, and DNA replication errors shape the spectra of genome changes in human skin'

*Response :* We have altered the title as suggested.

2. Abstract: usually ‘mutation load’ is used as singular Abstract: ‘…DNA replication stalling at common fragile sites Is a potent source…'Abstract, last sentence: please change ‘intrinsic factors’ -> ‘endogenous factors’

*Response:* The changes have been made to the abstract.

3. P. 3, line 39: ‘Large scale’ -> ‘Large-scale’

*Response:* We have fixed this term here and across the manuscript.

4. P. 3, line 44: there should be no comma after ‘Since’

*Response:* The comma has been removed.

5. P. 4, line 57: add space after ‘Pol’ and before the greek letter of the polymerase

*Response:* The space has been added.

6. P. 4, line 59: ‘Error-prone’

*Response:* This has been fixed here and across the manuscript.

7. P. 4, line 69: ‘UV radiation’

*Response:* This phrase has been altered as suggested.

8. P. 4, line 71: add comma after ‘melanocytes’

*Response:* The comma has been added.

9. P. 4, line 76: ‘burdens’ -> ‘burden’; ‘sun shielded’ -> ‘sun-shielded’

*Response:* These terms have been changed here and across the manuscript.

10. P. 5, line 82: add comma after ‘skin’, change ‘signature’ -> ‘mutation signature’

*Response:* The change has been made in the text.

11. P. 5, line 86: add comma after ‘(InDels)’

*Response:* The change has been made in the text.

12. P. 5, line 89: change to ‘…due to either small sample sizes or difficulties…’

*Response:* The change has been made in the manuscript.

13. P. 6, line 101: add comma after ‘errors’

*Response:* We have added a comma in this location.

14. P. 6, line 114: remove ‘-‘ after ‘cell’

*Response:* The change has been made in the text.

15. P. 6, line 116: here and elsewhere in the paper spell out all numbers below 10

*Response:* We have spelt out the numbers below 10 across the manuscript where feasible.

16. P. 6, lines 118 and 121: ‘whole-genome’
17. P. 7, line 122: ‘whole-genome’

*Response:* “whole-genome” has now replaced “whole genome” across the manuscript.

18. Figures 1-3, 5: ‘R-square’ -> ‘R-squared’

*Response:* The suggested changes to the figures have been made.

19. P. 7, line 135: ‘sequencing coverage’ -> ‘sequencing depth’ (was this average per site?)

*Response:* The text has been altered to clarify that the coverage denotes the average coverage per site .

Lines 173-174 - “The median sequencing depth for the samples was 78X with a minimum average coverage per site of 50X (S1 Table).

20. P. 7, line 137: ‘variations’ -> ‘variants’

*Response:* The change has been made here and across the manuscript.

21. P. 8, line 163: ‘see’ -> ‘saw’

*Response:* The change has been made in the text.

22. P. 9, line 169: add comma after ‘SBS5’

*Response:* The suggested change has been made in the text.

23. P. 9, line 173: ‘that’ -> ‘this’

*Response:* The suggested change has been made in the text.

24. P. 9, line 179: ‘SBS1-associated’

*Response:* The suggested change has been made in the text.

25. P. 10, line 189: please add a reference after ‘have previously been shown to be associated with UV-induced DNA damage in human cells’

*Response:* The references have now been added.

26. P. 10, line 206: ‘Whole-exome’

*Response:* The term “whole exome” has been replaced by “whole-exome” across the manuscript.

27. P. 12, line 240: remove comma after ‘Since’

*Response:* The suggested change has been made.

28. P. 12, line 247: ‘spanning 5 nucleotides or larger’ -> ‘spanning five or more nucleotides’

*Response:* The suggested change has been made across the manuscript.

29. P. 12, line 248: ‘have microhomology of one or more bases’

*Response:* The suggested change has been made across the manuscript.

30. P. 12, line 252: ‘deletions of 5 bases or more’ -> ‘deletions of five or more bases’

*Response:* The suggested change has been made across the manuscript.

31. P. 13, line 254: ‘spanning five or more nucleotides’

*Response:* The suggested change has been made across the manuscript.

32. P. 13, line 256: ‘indel load'

*Response:* The terms – “mutation loads” and “indel loads” have been altered to “mutation load” and “indel load” across the manuscript.

33. P. 13, line 257, and elsewhere in the manuscript: ‘InDel’ -> ‘indel’

*Response:* We have made the replacement across the manuscript.

34. P. 14, line 290 and elsewhere in the manuscript: ‘gender’ -> ‘sex’

*Response:* The term “gender” has now been replaced with “sex” in the manuscript.

35. P. 14, line 297: ‘do see’ -> ‘did see’

*Response:* The suggested change has been made in the manuscript.

36. P. 16, line 324: ‘whole-genome’

*Response:* “whole-genome” has now replaced “whole genome” across the manuscript.

37. P. 16, lines 327 and 338: add space before ‘bp’ and ‘Mbp’

*Response:* The change has been made in the text.

38. P. 17, line 351: ‘surmise -> ‘hypothesize’

*Response:* The suggested change has been made in the text.

39. P. 18, line 356: ‘Whole-genome’

*Response:* “whole-genome” has now replaced “whole genome” across the manuscript.

40. P. 18, line 373: ‘endogenously operating’ -> ‘endogenous’

*Response:* The term “operating” has been removed in this sentence.

41. P. 20, line 416: ‘loads’ -> ‘load’

*Response:* The terms – “mutation loads” and “indel loads” have been altered to “mutation load” and “indel load” across the manuscript.
